# MTF-Net: a brain tumor segmentation network with multi-scale transformer and feature fusion enhancement

**DOI:** 10.3389/fmed.2026.1934161

**Published:** 2026-10-26

**Authors:** Wenyan Liu, Yajie Cao, Yi Liu, Pengcheng Zhang, Zhiguo Gui, Lili Liu

**Affiliations:** 1 School of Information and Communication Engineering, North University of China, Taiyuan, China; 2 School of New Energy, Inner Mongolia University of Technology, Ordos, China

**Keywords:** brain tumor, feature enhancement, feature fusion, multi-scale transformer, segmentation

## Abstract

To address the issues of insufficient multimodal information fusion and inadequate long-range feature extraction in brain tumor segmentation, we propose a brain tumor segmentation network based on multi-scale Transformer and feature fusion enhancement (MTF-Net). In the second to fourth encoding layers of MTF-Net, the multi-scale and spatially-aware Transformer module (MSFormer) effectively integrates multi-scale deep convolution with spatially-aware MLP to perform local-global joint modeling under different receptive fields. Additionally, the last layer of the encoder employs a Channel-Enhanced Bi-level Attention Module (CEBAM), which combines a dual-layer sparse attention mechanism with a channel enhancement structure to enhance the capability of expressing long-range dependencies. Then, in the bridging section of the encoder-decoder, a Cross-Modal Feature Interaction Module (CFIM) is used to guide the fusion of features from MSFormer and the convolutional branch, improving boundary perception and consistency across modalities. Finally, we uses a hybrid loss function composed of binary cross entropy (BCE) and Dice Loss to alleviate the issue of class imbalance. Experimental results on the BraTS2019 brain tumor dataset show that the model achieves average Dice coefficients of 0.926, 0.921, and 0.896 for whole tumor, tumor core, and enhancing tumor, respectively. Experimental results on the BraTS2021 brain tumor dataset show that the model achieves average Dice coefficients of 0.918, 0.923, and 0.901 for whole tumor, tumor core, and enhancing tumor, respectively. Compared with the evaluated segmentation methods, the proposed model achieves competitive segmentation performance across the three tumor subregions.

## 1 Introduction

Brain tumors, especially gliomas, are highly aggressive malignant tumors that pose significant threats to the central nervous system and severely affect patients’ cognitive and neurological functions (1). Magnetic Resonance Imaging (MRI) is a non-invasive imaging technique that collects and images hydrogen nuclei signals using the magnetic resonance phenomenon. It has been widely used in clinical detection, classification, and postoperative evaluation of brain tumors (2). Multiple MRI modalities can provide complementary tissue contrast information, helping doctors more accurately identify and analyze tumor regions (3). However, traditional manual segmentation relies on expert experience, is inefficient and highly subjective, and fails to meet clinical needs. Therefore, in recent years, deep learning has been widely used for automatic segmentation of medical images. The proposals of Fully Convolutional Networks (FCN) (4) and U-Net (5) have significantly promoted the development of brain tumor segmentation technology.

Although Convolutional Neural Networks (CNNs) perform excellently in medical image segmentation, their limited receptive fields struggle to address the heterogeneity, blurry boundaries, and complex morphology of brain tumors. Transformer structures, with their strong global modeling capabilities, have been introduced to this field, such as in TransUNet (6) and Swin-Unet (7). However, Transformers typically have high computational costs and large memory consumption, making them challenging for practical application in computational medical image analysis. Moreover, brain tumor regions are often small in volume and variable in shape, and traditional attention mechanisms like SE (8) and CBAM (9) can enhance feature expression but suffer from parameter redundancy and insufficient spatial modeling capabilities. Despite the extensive exploration of multi-scale feature modeling, Transformer-based attention, and feature fusion strategies in medical image segmentation, existing methods often focus on improving these aspects independently. The effective integration of multi-scale spatial representation, efficient global dependency modeling, and feature interaction remains challenging, especially for heterogeneous brain tumor segmentation.

Existing brain tumor segmentation methods still face several challenges, including insufficient modeling of heterogeneous tumor structures, limited long-range dependency representation, and inadequate feature interaction among different semantic levels. Therefore, MTF-Net is designed to address these limitations by integrating multi-scale spatial modeling, efficient global dependency learning, and adaptive feature interaction within a unified framework.

To address these challenges, we propose a brain tumor segmentation network based on multi-scale Transformer and feature fusion enhancement (MTF-Net). Different from simply combining existing CNN, Transformer, and fusion components, MTF-Net introduces a hierarchical feature interaction strategy, where multi-scale feature extraction, sparse global attention, and cross-feature fusion are progressively integrated to enhance tumor representation. Specifically, the proposed framework combines multi-scale spatial modeling and Transformer-based global dependency learning to improve contextual feature representation, while the feature fusion mechanism further reduces semantic differences between different feature representations and enhances boundary perception.

The key contributions of this paper are as follows:

1. We propose a hierarchical feature interaction framework for multimodal brain tumor segmentation. Different from existing hybrid architectures that independently combine CNN and Transformer components, MTF-Net establishes a progressive interaction pathway among multi-scale spatial representation, sparse global dependency modeling, and cross-branch feature fusion.
2. We introduce the Multi-Scale Spatially-Aware Transformer module (MSFormer), which effectively captures both local and global features under different receptive fields by combining multi-scale depthwise convolution with a spatially-aware MLP in a parallel structure. Unlike conventional Transformer feed-forward networks, the spatially-aware MLP introduces depthwise convolution before pointwise channel projection to preserve spatial structural information. MSFormer is deployed at the intermediate encoding stages, where sufficient spatial resolution is retained for multi-scale structural and contextual modeling. This design improves the representation of complex and highly heterogeneous tumor regions.
3. To address the limited long-range dependency modeling and parameter redundancy of conventional Transformer structures in brain tumor segmentation, we introduce the Channel-Enhanced Bi-level Attention Module (CEBAM). Although CEBAM adopts the Bi-Level Routing Attention mechanism from BiFormer, it introduces a 3 × 3 depthwise separable convolution before BRA to incorporate local structural information and integrates the spatially-aware MLP after BRA to enhance spatial–channel interactions. CEBAM is applied only at the deepest encoding stage, where it combines local feature enhancement with sparse global semantic modeling on a reduced-resolution feature map.
4. Traditional U-Net-like segmentation networks generally transfer encoder features to the decoder through direct concatenation or addition. To improve the interaction between complementary feature representations, we design the Cross-Modal Feature Interaction Module (CFIM). Rather than independently weighting the four original MRI modalities, CFIM adaptively fuses the contextual features generated by MSFormer and the local structural features preserved by the parallel ConvBlock branch. It generates content-dependent fusion weights through coordinate attention and Softmax normalization, refines the two feature streams through symmetric multi-path convolutional branches, and performs adaptive cross-branch fusion. This design helps reduce the semantic discrepancy between the encoder and decoder and improves boundary-structure representation.

## 2 Related work

To analyze the existing brain tumor segmentation methods, we reviews them from three perspectives: convolutional neural networks (CNNs) and their variants, Transformer-based methods, and attention mechanisms with feature modeling.

### 2.1 Convolutional neural networks and related models

Convolutional Neural Networks (CNNs) have been widely applied in medical image segmentation tasks due to their capabilities in local perception and feature extraction. A series of encoder–decoder architectures, represented by U-Net (5) and its variants such as U-Net++ (10), DenseUNet (11), and Attention U-Net (12), integrate shallow details with deep semantic features through skip connections, achieving remarkable performance in automatic brain tumor segmentation. To enhance the modeling capacity for multi-scale information, researchers have proposed modules like Inception (13) and Atrous Spatial Pyramid Pooling (ASPP) (14), which are designed to capture contextual features under different receptive fields. Agarwal et al. (35) proposed a multi-scale dual-channel feature embedding decoder that combines parallel CNN encoders with attention-gated Swin Transformers to capture both short- and long-range dependencies at multiple scales. This work demonstrates the effectiveness of multi-scale feature embedding and hierarchical attention in biomedical image segmentation. MobileNets (15) and EfficientNet (16) focus on network simplification and computational efficiency by employing depthwise separable convolutions and compound scaling strategies, thereby enabling lightweight modeling. However, the fixed receptive fields of traditional CNNs limit their ability to model long-range dependencies, which presents challenges, especially in tumor regions with fuzzy boundaries and high heterogeneity. To address this limitation, we incorporate the MSFormer module based on convolutions, which integrates a Multi-Scale Depthwise Convolution (MSDC) module with a Spatially-Aware MLP (SAMLP) in a parallel structure, thereby enhancing global context modeling while maintaining sensitivity to local features.

### 2.2 Transformer-based methods

Transformer architectures have been increasingly introduced into medical image analysis in recent years due to their powerful global modeling capabilities. Vision Transformer (ViT) (17) maps image slices into a sequence of patches for processing. TransUNet (6) combines the strengths of CNNs and ViT to achieve joint modeling of local structures and long-range dependencies in medical images, proposed by Chen et al. Subsequent models such as Swin Transformer (18) and Pyramid Vision Transformer (PVT) (19) implement hierarchical modeling through shifted window attention and pyramid structures, respectively, thereby alleviating the computational burden of standard fully-connected attention. MaxViT (20) further integrates local and global grid attention mechanisms to jointly model fine-grained details and contextual information across multiple scales. Datta et al. (36) proposed a unified student–teacher framework that integrates SwinPAC-UNETR-based tumor segmentation with a multimodal Transformer for survival prediction, using exponential moving average and knowledge distillation to stabilize training and reduce error accumulation across tasks. However, the attention modules and feed-forward networks in Transformer architectures still suffer from parameter redundancy and insufficient structural awareness of visual data. These limitations are particularly challenging when modeling heterogeneous lesions such as brain tumors. To address this, we designs the CEBAM module, which, based on BiFormer (21), integrates a dual-layer sparse attention mechanism (BRA) with channel modeling structures and introduces spatially-aware MLP, effectively modeling deep semantic relationships and global structural features.

### 2.3 Attention mechanisms and feature modeling

Attention mechanisms are widely employed in medical image segmentation to guide models toward critical regions, thereby mitigating feature redundancy and enhancing semantic representation. The SE (8) and CBAM (9) improve the quality of feature representation from the channel dimension and the joint spatial–channel perspective, respectively, and have been adopted in various segmentation networks. Brain tumor MRI multimodal images are highly complementary, but traditional attention mechanisms often focus on single-modal or single-path modeling, lacking the ability to capture the complementarity of features across multimodal inputs. During the decoding process, CFIM fuses features from both the MSFormer and convolutional branches, while integrating Coordinate Attention (CA) (22) to perform spatial and channel-wise saliency reweighting. Moreover, multi-path convolution is employed to refine fusion accuracy, thereby enhancing the network's ability to preserve structural boundaries and ensure modality alignment.

## 3 Methodology

### 3.1 MTF-Net network

We propose a brain tumor segmentation network based on multi-scale transformer attention and feature fusion enhancement (MTF-Net). The overall architecture of MTF-Net is illustrated in Figure 1.

**Figure 1:**
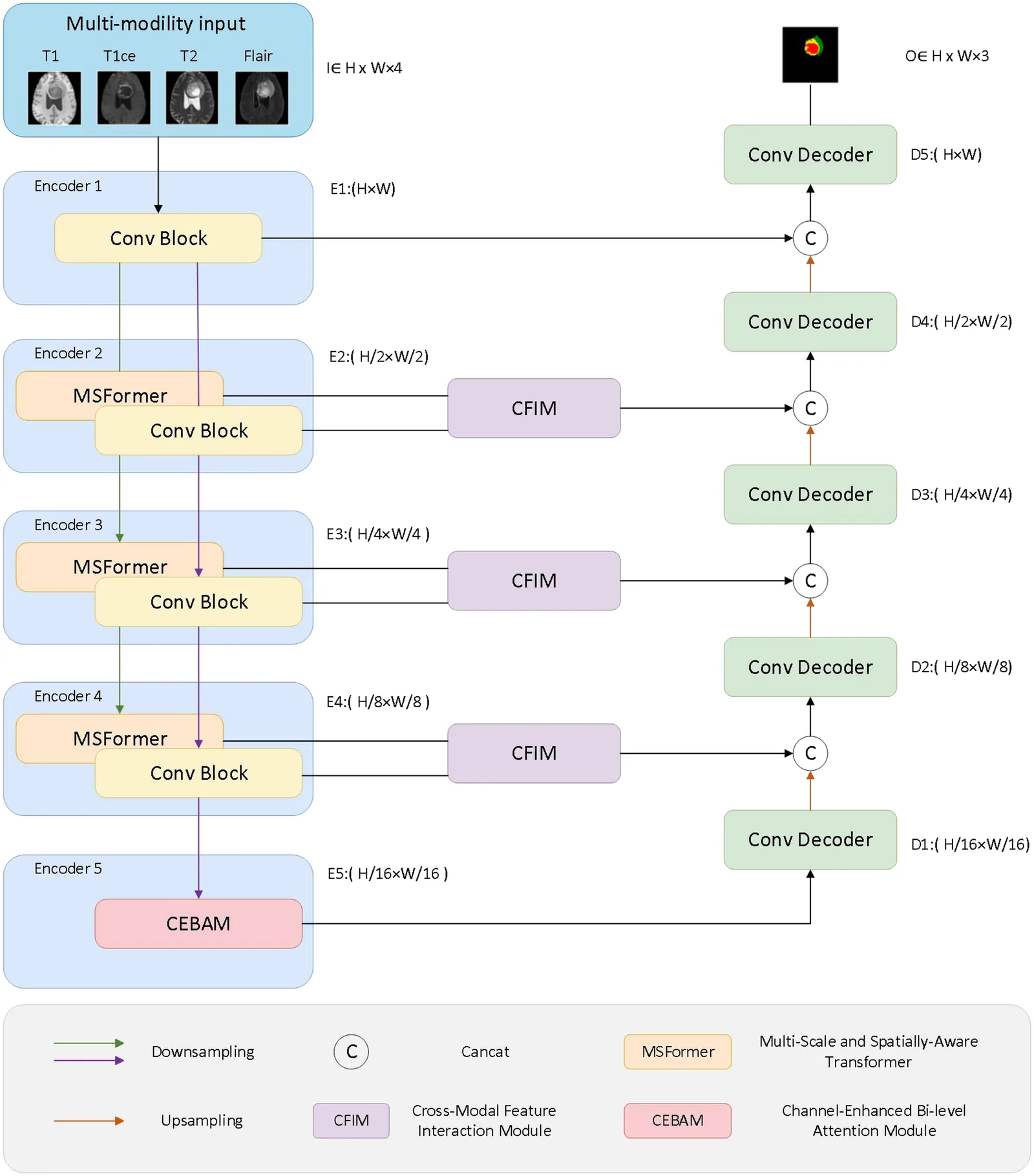
Overall architecture of the proposed MTF-Net.

In the encoder section, the first layer uses a convolutional module (Conv Block) to extract low-level local features such as edge textures. In the second to fourth layers, the proposed MSFormer is introduced, running in parallel with the convolutional branch, to enhance the model's ability to model context at different receptive fields and spatial positions. The final layer employs the CEBAM to globally model the entire feature map, further enhancing the network's global perception and long-range dependency modeling capabilities. To mitigate the semantic gap between the encoder and decoder, MTF-Net uses CFIM in the skip connections to fuse and enhance the features output from different branches, strengthening the representation of boundary areas and regions with modal differences. In the decoder, a multi-layer convolutional decoder (Conv Decoder) is used for progressively upsampling to restore spatial resolution. MTF-Net enhances the model's ability to recognize heterogeneous tumor structures and preserve boundary details through multi-scale modeling, sparse global attention, and cross-modal feature fusion. Through this hierarchical architecture, MTF-Net progressively integrates multi-scale spatial representation, sparse global dependency modeling, and cross-branch feature interaction at different network stages, enabling a better balance between local boundary preservation and global contextual understanding.

### 3.2 MSFormer module

Convolution-based encoder structures have limitations in modeling long-range dependencies due to their constrained receptive fields, making it difficult to capture global context information across regions. To address this, Transformer models have gradually been introduced into medical image segmentation tasks. However, the classical Transformer architecture suffers from high computational complexity and significant parameter redundancy. The standard feed-forward neural network (FFN) used in Transformers lacks structural awareness when processing image spatial features, making it challenging to handle complex boundaries and irregularly structured lesion areas. To balance global modeling capabilities with spatial structure modeling, we proposes a Multi-Scale and Spatially-Aware Transformer (MSFormer), as shown in Figure 2. As a key component in the second to fourth layers of the encoder, this module collaboratively models local structures, spatial position information, and long-range dependency semantics through a Multi-Scale Depthwise Convolution (MSDC) module and a Spatial-Aware Multi-Layer Perceptron (SAMLP).

**Figure 2:**
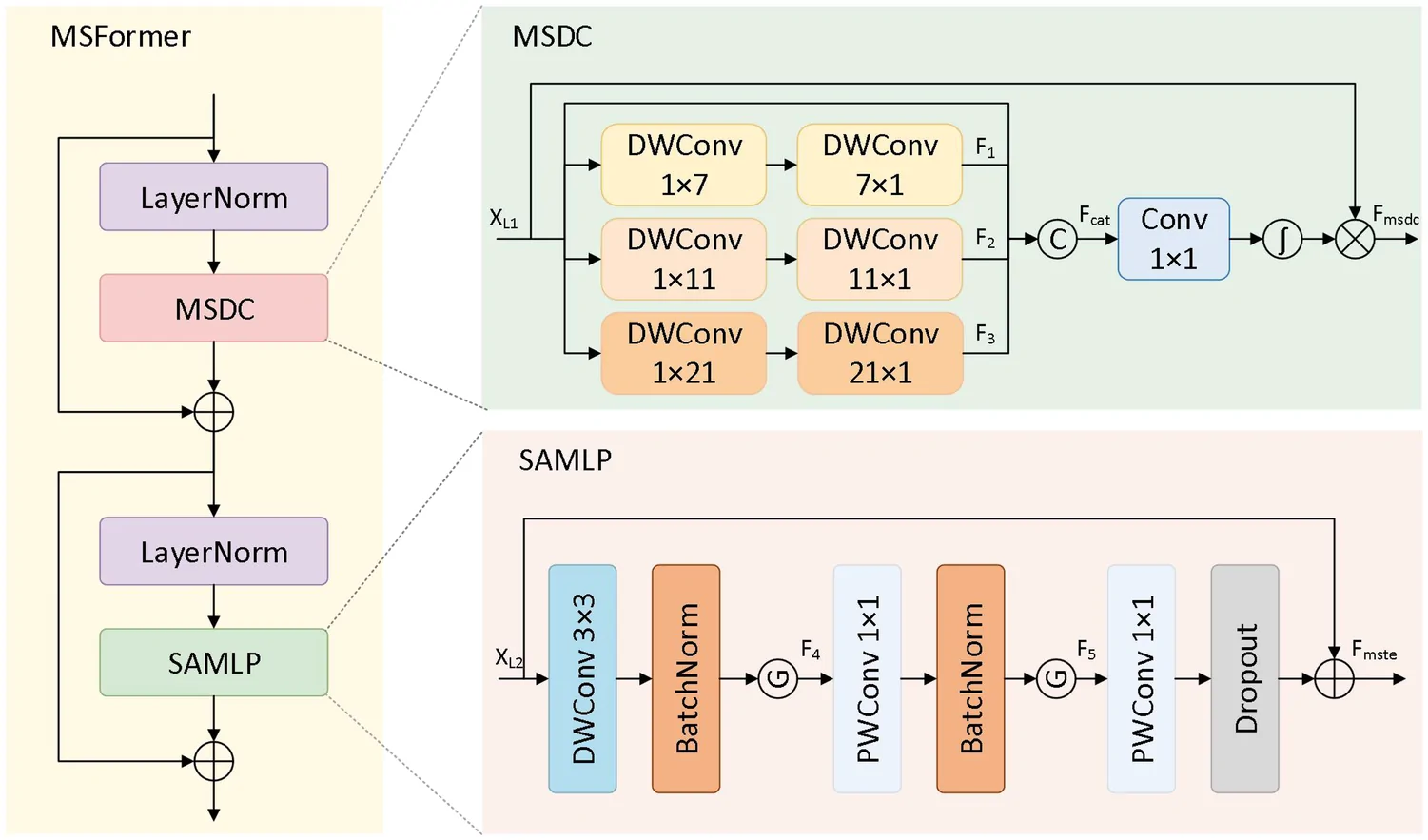
Architecture of the MSFormer module.

#### 3.2.1 MSDC module

The MSDC module performs multi-scale feature extraction through multiple sets of depthwise convolutions (DWConv). These convolution operations enable the network to handle different receptive fields and capture spatial features at various scales. By concatenating features along the channel dimension, this module achieves multi-receptive field spatial modeling, ensuring that the network can learn both local and global spatial patterns in the tumor region, thereby enhancing the segmentation accuracy of the tumor area.

Specifically, given an input feature map $X_{L1}\in R^{C\times H\times W}$, three scales of features are extracted using three parallel DWConv branches, as defined in (Equations 1–3).

$$F_{1}=\mathrm{DWCon}v_{1\times 7}(\mathrm{DWCon}v_{7\times 1}(X_{L1}))$$ (1)

$$F_{2}=\mathrm{DWCon}v_{1\times 11}(\mathrm{DWCon}v_{11\times 1}(X_{L1}))$$ (2)

$$F_{3}=\mathrm{DWCon}v_{1\times 21}(\mathrm{DWCon}v_{21\times 1}(X_{L1}))$$ (3)

Subsequently, the above feature maps are concatenated along the channel dimension to form the fused feature $F_{\mathrm{cat}}$, as shown in (Equation 4).

$$F_{\mathrm{cat}}=\mathrm{Concat}(F_{1},F_{2},F_{3})$$ (4)

To reduce channel dimensionality and integrate cross-scale semantic information, a 1 × 1 convolution is employed for channel compression. The features are then scored using a Softmax function, and the resulting weights are multiplied by the initial features to generate the final output $F_{\mathrm{msdc}}$, as expressed in (Equation 5).

$$F_{\mathrm{msdc}}=X_{L1}\cdot \mathrm{Softmax}(\mathrm{Con}v_{1\times 1}(F_{\mathrm{cat}}))$$ (5)

Here, DWConv denotes depthwise separable convolution, Concat represents channel-wise concatenation, and Conv refers to a 1 × 1 convolution used for channel compression and feature mapping.

#### 3.2.2 SAMLP module

The SAMLP module is used to enhance the model's ability to model spatial position variations and channel importance. SAMLP strengthens the interaction between channels and spatial features through depthwise convolution and pointwise convolution (PWConv). A dropout operation is applied to improve the model's regularization capability, preventing overfitting. Finally, residual connections are used to fuse the enhanced features, enhancing the model's representation capability for complex tumor structures.

Specifically, the input feature $X_{L2}$ is first processed by a 3  × 3 depthwise convolution to extract local spatial structural information, followed by Batch Normalization and a GELU activation function, as defined in (Equation 6).

$$F_{4}=\mathrm{GELU}(\mathrm{BN}(\mathrm{DWCon}v_{3\times 3}(X_{L2})))$$ (6)

Then, a 1 × 1 pointwise convolution (PWConv) is applied to increase the channel dimensionality, followed by Batch Normalization and a GELU activation function, as shown in (Equation 7).

$$F_{5}=\mathrm{GELU}(\mathrm{BN}(\mathrm{PWCon}v_{1\times 1}(F_{4})))$$ (7)

Finally, a 1 × 1 PWConv is used to restore the channel dimensionality, followed by a Dropout operation to enhance regularization. The output is then combined with the original input via a residual connection, as defined in (Equation 8).

$$F_{\mathrm{samlp}}=X_{L2}+\mathrm{Dropout}(\mathrm{PWCon}v_{1\times 1}(F_{5}))$$ (8)

Here, DWConv denotes depthwise separable convolution, PWConv refers to pointwise convolution, BN stands for Batch Normalization, and GELU represents the Gaussian Error Linear Unit activation function.

### 3.3 CEBAM module

Although traditional CNN encoders are effective in modeling local structures, they still face challenges in modeling long-range dependencies and global semantic information in complex brain tumor images. While Transformers have the advantage of modeling long-range dependencies, their fully connected attention mechanism is typically computationally expensive and prone to parameter redundancy. BiFormer addresses this by proposing a Bi-Level Routing Attention (BRA) mechanism, which constructs a semantic map at the regional level to calculate the similarity between different regions and then selects the top k most relevant regions for token-level fine-grained modeling. By using a sparse attention mechanism, the model can enhance its global information perception while maintaining efficiency. This mechanism reduces redundant information, improving the model's efficiency in long-range dependency modeling. The CEBAM module designed in we introduces the Bi-Level Routing Attention (BRA) and incorporates a Spatial-Aware Multi-Layer Perceptron (SAMLP) module. CEBAM combines lightweight convolution operations with spatial awareness, effectively enhancing the interaction between channels and spatial features, enabling the model to capture deep semantic information at higher levels. Figure 3 illustrates the structure of the proposed CEBAM module.

**Figure 3:**
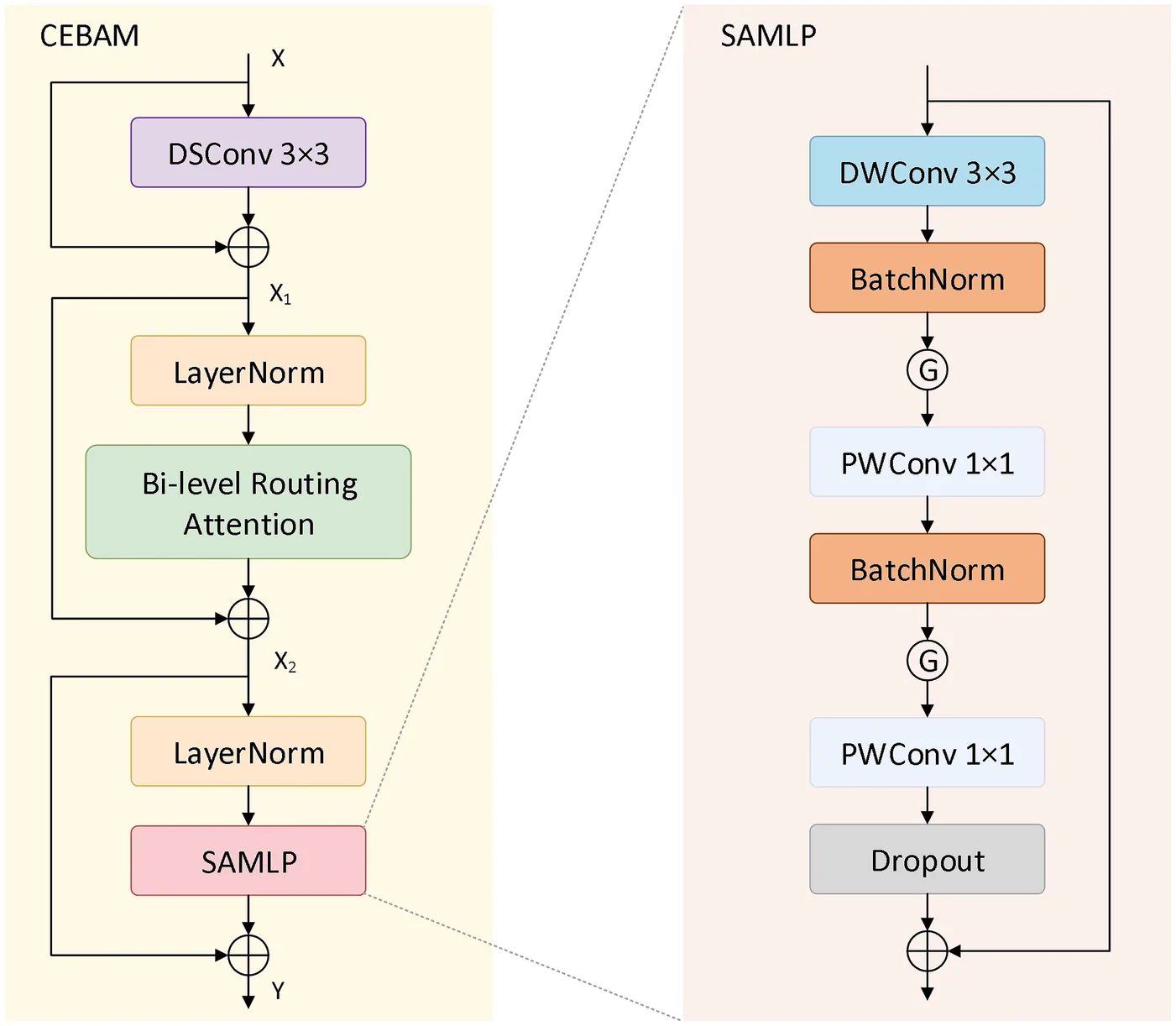
Architecture of the CEBAM module.

First, the input feature map *X* is processed by a 3 × 3 depthwise separable convolution to extract local spatial structural information such as edges and textures, resulting in the output feature $X_{1}$, as defined in (Equation 9).

$$X_{1}=X+\mathrm{DSCon}v_{3\times 3}(X)$$ (9)

Subsequently, Layer Normalization (LayerNorm) is applied for normalization, and the result is fed into the BRA module. The BRA module constructs a semantic graph at the region level by computing the similarity between regions and identifying the top-k most relevant region indices. Fine-grained token-level attention is then performed within these selected region sets to enable sparse global information modeling. A residual connection is used to retain the original information, resulting in the output feature $X_{2}$, as defined in (Equation 10).

$$X_{2}=X_{1}+\mathrm{BRA}(\mathrm{LN}(X_{1}))$$ (10)

Finally, to further enhance channel modeling capability, the SAMLP module is introduced. A residual connection is then employed to fuse the input and the enhanced features, yielding the final output *Y*, as defined in (Equation 11).

$$Y=X_{2}+\mathrm{SAMLP}(\mathrm{LN}(X_{2}))$$ (11)

Here, DSConv denotes the 3 × 3 depthwise separable convolution, LN refers to Layer Normalization, BRA stands for Bi-Level Routing Attention, and SAMLP represents the Spatial-Aware MLP.

### 3.4 CFIM module

In the multimodal brain tumor segmentation task, the structural details and semantic information contained in different modalities (such as T1, T1ce, T2, FLAIR) are highly complementary. To fully explore the information correlation between the convolutional branch and the MSFormer branch, we proposes the Cross-Modal Feature Interaction Module (CFIM), as shown in Figure 4. This module effectively reduces the semantic gap between the encoder and decoder by concatenating and fusing features from both branches. The fusion ratio is controlled by a weighting coefficient *α*, and the CFIM module improves the boundary accuracy and modality consistency of the tumor region while reducing the semantic gap. Multi-branch convolution and Coordinate Attention (CA) effectively enhance the feature fusion between different modalities by weighting the significance of both spatial and channel dimensions. By introducing coordinate information into the attention mechanism, the model can perform precise feature reweighting both spatially and channel-wise, optimizing multimodal information fusion, especially in maintaining tumor boundaries and ensuring modality alignment.

**Figure 4:**
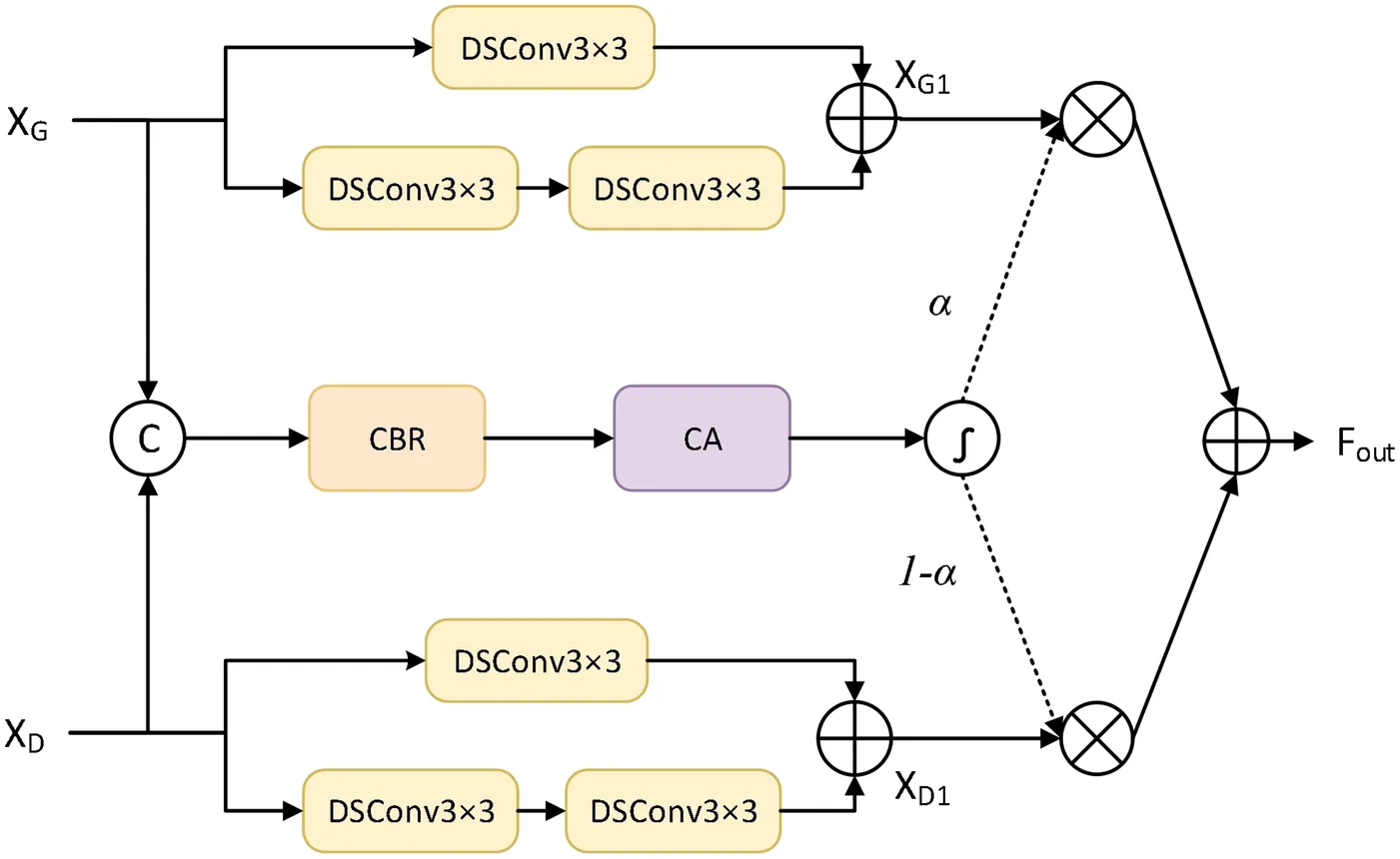
Structure of the CFIM module.

The input to this module consists of two feature maps: $X_{M}$, obtained from the output of the MSFormer module, and $X_{D}$, from the output of the convolutional block. First, these two features are concatenated and fed into a sequential branch composed of two consecutive CBR blocks (Conv 3 × 3 + BatchNorm + ReLU), one CA module, and a Softmax function, to generate an attention weight coefficient *α*. Next, $X_{M}$ and $X_{D}$ are separately passed through two symmetric multi-path convolutional branches for feature enhancement, resulting in $X_{G1}$ and $X_{D1}$, respectively. Each branch contains two paths: one consisting of a single 3 × 3 depthwise separable convolution, and the other comprising two stacked 3 × 3 depthwise separable convolutions. The outputs of these two paths are summed via a residual connection. The output feature $Y_{\mathrm{out}}$ of the module is obtained by multiplying $X_{G1}$ with *α*, multiplying $X_{D1}$ with (1 − *α*), and then summing the two results: This process is formulated in (Equations 12–15).

$$\alpha =\mathrm{Softmax}(\mathrm{CA}(\mathrm{CBR}(\mathrm{Concat}(X_{M},X_{D}))))$$ (12)

$$X_{G1}=\mathrm{DSCon}v_{3\times 3}(X_{G})+\mathrm{DSCon}v_{3\times 3}(\mathrm{DSCon}v_{3\times 3}(X_{G}))$$ (13)

$$X_{D1}=\mathrm{DSCon}v_{3\times 3}(X_{D})+\mathrm{DSCon}v_{3\times 3}(\mathrm{DSCon}v_{3\times 3}(X_{D}))$$ (14)

$$F_{out}=X_{G1}\;\times \;\alpha +X_{D1}\times (1-\alpha )$$ (15)

## 4 Experimental settings

### 4.1 Dataset

In this study, we utilize the publicly available datasets from the 2019 and 2021 Brain Tumor Segmentation (BraTS 2019 and BraTS 2021) competitions. The BraTS 2019 and BraTS 2021 datasets comprise multi-modal MRI images primarily designed for automated brain tumor segmentation tasks. The BraTS 2019 dataset comprises MRI images from 335 patients with glioma, including 76 cases of low-grade glioma (LGG) and 259 cases of high-grade glioma (HGG). Each case provides four MRI modalities: native T1-weighted MRI (T1), post-contrast T1-weighted MRI (T1ce), T2-weighted MRI (T2), and T2 fluid-attenuated inversion recovery MRI (FLAIR), together with expert-annotated tumor segmentation labels.

The BraTS 2021 benchmark contains two main tasks: brain tumor subregion segmentation and MGMT promoter methylation classification. In this study, only Task 1, namely brain tumor subregion segmentation, was considered. The complete BraTS 2021 benchmark comprises 2,040 cases, including 1,251 training cases, 219 validation cases, and 570 testing cases. Because the segmentation annotations of the official validation and testing cohorts were not publicly available, only the 1,251 labeled cases from the official BraTS 2021 training cohort were used in this study. The official BraTS 2021 benchmark paper was cited to provide the detailed dataset description and challenge protocol. The official benchmark paper and challenge website confirm the two tasks, the total cohort size, and the definitions of the evaluated tumor subregions.

Each BraTS 2021 case also contains four pre-operative MRI modalities: T1, T1ce, T2, and FLAIR. The original segmentation annotations comprise three tumor-tissue labels: label 1 represents the necrotic/non-enhancing tumor core (NCR/NET), label 2 represents the peritumoral edematous/invaded tissue (ED), and label 4 represents the enhancing tumor (ET). Based on these original annotations, the three composite tumor regions evaluated in this study were defined as follows:

1. Whole Tumor (WT): the union of labels 1, 2, and 4, including the necrotic/non-enhancing tumor core, peritumoral edema, and enhancing tumor;
2. Tumor Core (TC): the union of labels 1 and 4, including the necrotic/non-enhancing tumor core and enhancing tumor;
3. Enhancing Tumor (ET): label 4, representing the enhancing portion of the tumor.

These label values and composite evaluation regions are consistent with the official BraTS 2021 definitions.

For model development and evaluation, the complete BraTS 2019 cohort and the 1,251 labeled cases from the BraTS 2021 training cohort were independently divided into training, validation, and testing subsets at the patient level with a ratio of 7:1:2. Specifically, the patient-level partitioning was performed before any preprocessing operations and two-dimensional slice extraction to strictly prevent slice-level data leakage. Therefore, all slices generated from the same three-dimensional MRI volume were retained exclusively within one subset, and no slices from the same patient were shared among the training, validation, and testing sets. This strategy ensures that information from the same patient cannot appear in multiple subsets during model training and evaluation. The training subsets were used to optimize the network parameters. The validation subsets were used for hyperparameter selection, training-process monitoring, and model-checkpoint selection. The independent testing subsets were not used for hyperparameter tuning or model selection and were used only for the final quantitative and qualitative evaluation. Before conducting experiments, the patient identifiers of the BraTS 2019 and BraTS 2021 cohorts were compared to examine potential overlap between the two datasets. No overlapping patients were identified between the two cohorts. Therefore, BraTS 2019 and BraTS 2021 were treated as independent datasets in this study, and all experiments on the two benchmarks were conducted separately without any patient-level data sharing or cross-dataset information leakage.

The official BraTS validation and testing servers were not used in this study. Therefore, the reported results were obtained from the internally defined patient-level testing subsets and should be interpreted as internal experimental results rather than official BraTS leaderboard results.

### 4.2 Data preprocessing

The officially provided dataset consists of 3D MRI images with a unified pixel size of 240 × 240 × 155. These data are pre-operative MRI scans, and each case includes four types of brain MRI modalities: T1-weighted MRI (T1), T1-weighted MRI with Contrast Enhancement (T1ce), T2-weighted MRI (T2), and Fluid-Attenuated Inversion Recovery (FLAIR), as well as a tumor label image annotated by experts.

Directly using the officially provided 3D format images for training would lead to a long training cycle and easily cause high memory and GPU consumption. In order to reduce computational cost, improve training efficiency, and increase the amount of sample data, we adopts a method of converting 3D medical image slices into 2D images to fit the proposed model structure. The original 3D data size of each modality is 240 × 240 × 155. Considering that the original MRI volumes contain substantial redundant background information, directly using the complete 240 × 240 × 155 volumes would increase computational cost and foreground-background imbalance. Therefore, a center-cropping strategy was adopted to reduce redundant regions and improve training efficiency. Before preprocessing, tumor masks were analyzed to verify the coverage of the cropping window. The results confirmed that all WT, TC, and ET regions were completely contained within the central 160 × 160 region, ensuring that no tumor-related information was excluded. After cropping, each volume was converted into 155 two-dimensional slices of 160 × 160 pixels for the proposed 2D segmentation framework.

Specifically, the crop center was set to the geometric center of each axial image. For the original in-plane size of 240 × 240 pixels, the index ranges [40, 200) along both spatial dimensions were retained, resulting in a fixed crop size of 160 × 160 pixels. The complete through-plane dimension of 155 slices remained unchanged. Identical cropping coordinates were applied to the four MRI modalities and the corresponding segmentation label to preserve their spatial alignment. The cropping operation was implemented through direct array indexing without spatial resizing; therefore, no image interpolation or label resampling was performed. Tumors located near the crop boundary were handled through the above-mentioned mask-coverage verification before preprocessing.

The 155 axial slices generated from each cropped volume were treated as candidate slices. During sample generation, slices containing at least one nonzero tumor label were retained, whereas slices without annotated tumor regions were excluded. For each retained slice, the four modalities were stacked in the order of FLAIR, T1, T1ce, and T2, and the corresponding segmentation label was saved using the same spatial coordinates.

Due to the differences in imaging mechanisms among different modalities, the image contrast also varies. In order to effectively balance the data intensity between different modalities and enable the model to better capture and utilize the complementary information among modalities, we adopts a data normalization operation, as shown in (Equation 16).

$$z=\frac{x-\bar{x}}{\sigma }$$ (16)

In the equation, *x* represents the cropped 2D slice image, $\bar{x}$ is the mean of the input image, σ is the standard deviation of the input image, and *z* denotes the image after normalization.

In the implementation, the intensity values of each MRI modality were first clipped to the 1st and 99th percentiles to reduce the influence of extreme values. The mean and standard deviation used for normalization were calculated from the nonzero voxels of the corresponding modality. After normalization, the minimum normalized intensity was assigned a value of −9 to distinguish the background region. No additional skull-stripping operation was performed, and the MRI data were processed in the form provided by the BraTS datasets.

No random flipping, rotation, scaling, elastic deformation, or intensity perturbation was applied during the offline data preprocessing stage.

As shown in Figure 5, a 2D slice of a certain case after the preprocessing is presented, from left to right: T1, T1ce, T2, FLAIR, and GT (ground truth) label. In both the network prediction map and the ground truth, the green region represents the whole tumor area, the yellow region indicates the enhancing tumor area, and the red region corresponds to the necrotic and non-enhancing tumor area.

**Figure 5:**
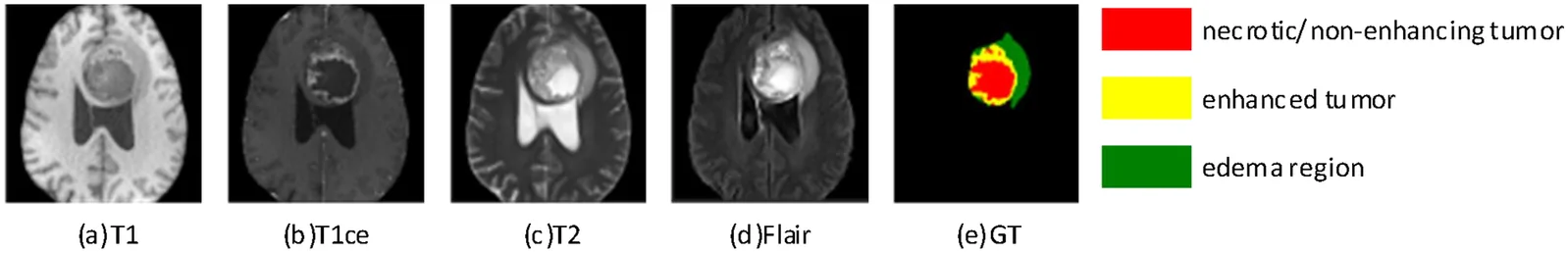
Images after data preprocessing from left to right as **(a)** T1, **(b)** T1ce, **(c)** T2, **(d)** flair, and **(e)** GT label.

### 4.3 Experimental environment configuration

All experiments are conducted using the deep learning framework PyTorch 1.11.0 with CUDA 11.2. The hardware environment includes a CPU with 80 vCPUs (AMD EPYC 7,642 48-Core Processor), 30 GB of memory, and a GPU of NVIDIA RTX 3,090 with 24 GB of VRAM. During training, the Adaptive Moment Estimation (Adam) algorithm is selected as the optimizer to update the network weights. The initial learning rate is set to 0.001, the momentum parameter is 0.9, and the weight decay coefficient is 1 × 10^−5^. The batch size is set to 16, and the total number of training epochs is 50. No mixed precision training was used, and all experiments were conducted using standard FP32 precision. Since MTF-Net adopts a 2D slice-based segmentation strategy, no 3D patch sampling was applied. All cropped axial slices were directly used for training. Class-balanced sampling was not employed; instead, the foreground-background imbalance was mitigated through the combination of BCE loss and Dice loss. During inference, no additional post-processing operations were performed, and the final segmentation masks were directly generated from network predictions.

To ensure a fair comparison between MTF-Net and existing 2D and 3D brain tumor segmentation methods, all comparative experiments were conducted under a unified experimental protocol. All baseline methods presented in Tables 1, 2 were reimplemented by the authors under the same experimental framework. None of the reported baseline results were directly obtained from previous publications. For all methods, the same patient-level data partitions were adopted before slice extraction to prevent potential data leakage between training, validation, and testing sets. Meanwhile, identical MRI modalities, tumor-label definitions, preprocessing and intensity-normalization procedures, hardware and software environments, fixed random seed of 26, optimizer settings, learning-rate schedule, number of training epochs, and evaluation metrics were used. No additional post-processing was applied to any method.

**Table 1:** Comparative experiments of advanced networks on BraTS2019 dataset.

Models	Dice↑			Precision↑			Sensitivity↑			Hausdorff95(mm) ↓		
	WT	TC	ET	WT	TC	ET	WT	TC	ET	WT	TC	ET
U-net (5)	0.836	0.816	0.756	0.860	0.847	0.770	0.878	0.900	0.830	5.684	5.925	3.804
U-net++ (10)	0.845	0.845	0.776	0.870	0.881	0.880	0.866	0.913	0.818	5.606	5.792	3.660
DenseUnet (11)	0.845	0.866	0.780	0.867	0.906	0.793	0.874	0.909	0.837	5.600	5.770	3.574
TransUnet (6)	0.852	0.834	0.810	0.875	0.934	0.915	0.873	0.909	0.896	5.570	5.545	3.490
TransBTSv2 (24)	0.902	0.848	0.802	0.852	0.893	0.789	0.902	0.922	0.860	5.432	5.473	3.696
nnU-Net (31)	0.891	0.852	0.785	0.919	0.911	0.792	0.884	0.852	0.825	5.562	5.641	3.624
Mmformer (25)	0.900	0.817	0.690	0.885	0.858	0.765	0.925	0.829	0.779	5.771	6.333	4.787
MMMViT (26)	0.900	0.822	0.722	0.894	0.867	0.793	0.918	0.825	0.772	5.994	6.650	3.524
SwinUNETR-V2 (32)	0.903	0.898	0.873	0.922	0.908	0.887	0.910	0.883	0.859	5.282	5.438	3.384
Fed-MUnet (27)	0 . 902	0 . 895	0.887	0.937	0.914	0.935	0.881	0.891	0.851	5.721	6.147	**3** **.** **149**
N-LiNet (33)	0.910	0.895	0.872	0.911	0.910	0.898	0.890	0.881	0.887	5.434	5.482	3.418
CFNet (28)	0.913	0 . 908	0 . 892	0.939	0.926	0.934	0.896	0.893	0.862	5.547	5.667	3.639
GHyperFormer (29)	0.910	0.894	0.872	0.928	0.918	0.903	0.917	0.904	0.869	5.583	5.929	3 . 685
AttCo (34)	0.917	0.903	0.866	0.924	0.922	0.920	0.910	0.901	0.894	5.389	5.367	3.256
Ours(MTF-Net)	**0** **.** **926**	**0** **.** **921**	**0** **.** **896**	**0** **.** **942**	**0** **.** **943**	**0** **.** **944**	**0** **.** **927**	**0** **.** **939**	**0** **.** **926**	**5** **.** **123**	**5** **.** **310**	3.203

Bold values indicate the best results. Underlined values indicate the second-best results.

**Table 2:** Comparative experiments of advanced networks on BraTS2021 dataset.

Models	Dice↑			Precision↑			Sensitivity↑			Hausdorff95(mm) ↓		
	WT	TC	ET	WT	TC	ET	WT	TC	ET	WT	TC	ET
U-net (5)	0.849	0.837	0.768	0.885	0.894	0.801	0.861	0.875	0.804	5.634	5.643	3.910
U-net++ (10)	0.858	0.839	0.783	0.870	0.899	0.815	0.872	0.885	0.820	5.574	5.663	3.831
DenseUnet (11)	0.857	0.844	0.781	0.892	0.900	0.812	0.872	0.890	0.821	5.575	5.616	3.844
TransUnet (6)	0.857	0.851	0.773	0.900	0.906	0.804	0.865	0.891	0.812	5.558	5.600	4.258
nnUnet (31)	0.888	0.851	0.796	0.910	0.902	0.808	0.871	0.861	0.834	5.557	5.603	3.493
Mmformer (25)	0.900	0.817	0.794	0.898	0.883	0.828	0.873	0.856	0.812	5.771	6.333	4.787
SwinUNETR-V2 (32)	0.910	0.900	0.878	0.922	0.909	0.891	0.902	0.886	0.862	5.560	5.420	3.370
Fed-MUnet (27)	0.900	0.891	0.860	0.927	0.912	**0** **.** **935**	0.879	0.879	0.864	5.724	5.672	3.361
N-LiNet (33)	0.910	0 . 897	0 . 875	0.912	0 . 911	0 . 901	0.892	0 . 884	0 . 878	5 . 410	5 . 460	3.390
LATUP-Net (30)	0.902	0.895	0.839	0.914	0.929	0.915	0.883	0.913	0.852	5.535	5.645	3.637
GHyperFormer (29)	**0** **.** **919**	0.899	0.867	**0** **.** **928**	0.941	0.919	**0** **.** **914**	0.917	0.866	5.527	5.429	3.641
AttCo (34)	0.916	0.908	0.872	0.925	0.924	0.913	0.909	0.899	**0** **.** **892**	5.382	5.372	3.365
Ours(MTF-Net)	0.918	**0** **.** **923**	**0** **.** **901**	0.924	**0** **.** **953**	0.921	0.908	**0** **.** **930**	0.884	**5** **.** **375**	**5** **.** **344**	**3.357**

Bold values indicate the best results. Underlined values indicate the second-best results.

For the 2D methods, the same retained axial slices were used as input. For the 3D methods, the corresponding three-dimensional volumes from the same patient subsets were used to preserve their original volumetric structures. Although the input organization followed the inherent requirements of different architectures, including slice-based inputs for 2D methods and volumetric inputs for 3D methods, the dataset partitioning, preprocessing principles, training conditions, and evaluation protocols were consistently maintained. Therefore, the comparison between 2D and 3D methods reflects the performance differences among network architectures under the same experimental environment.

To improve experimental reproducibility, the random seed was fixed at 26 for data processing and model training. All quantitative results reported in this study were obtained from a single run under this fixed-seed setting.

In addition, to further improve the stability of model optimization, a Cosine Annealing learning rate schedule (23) is adopted to dynamically reduce the learning rate and ensure that the network converges more smoothly to a global optimum. The learning rate schedule is shown in Figure 6. The training and validation losses were monitored throughout the 50 training epochs, as shown in Figure 7. Model selection was performed exclusively on the validation subset, and the checkpoint corresponding to the lowest validation loss was retained for final testing. Under the reported experimental setting, the selected checkpoint was obtained at epoch 48. The testing subset was not accessed during training, hyperparameter selection, or checkpoint selection. The detailed training experiment configuration is shown in Table 3.

**Figure 6:**
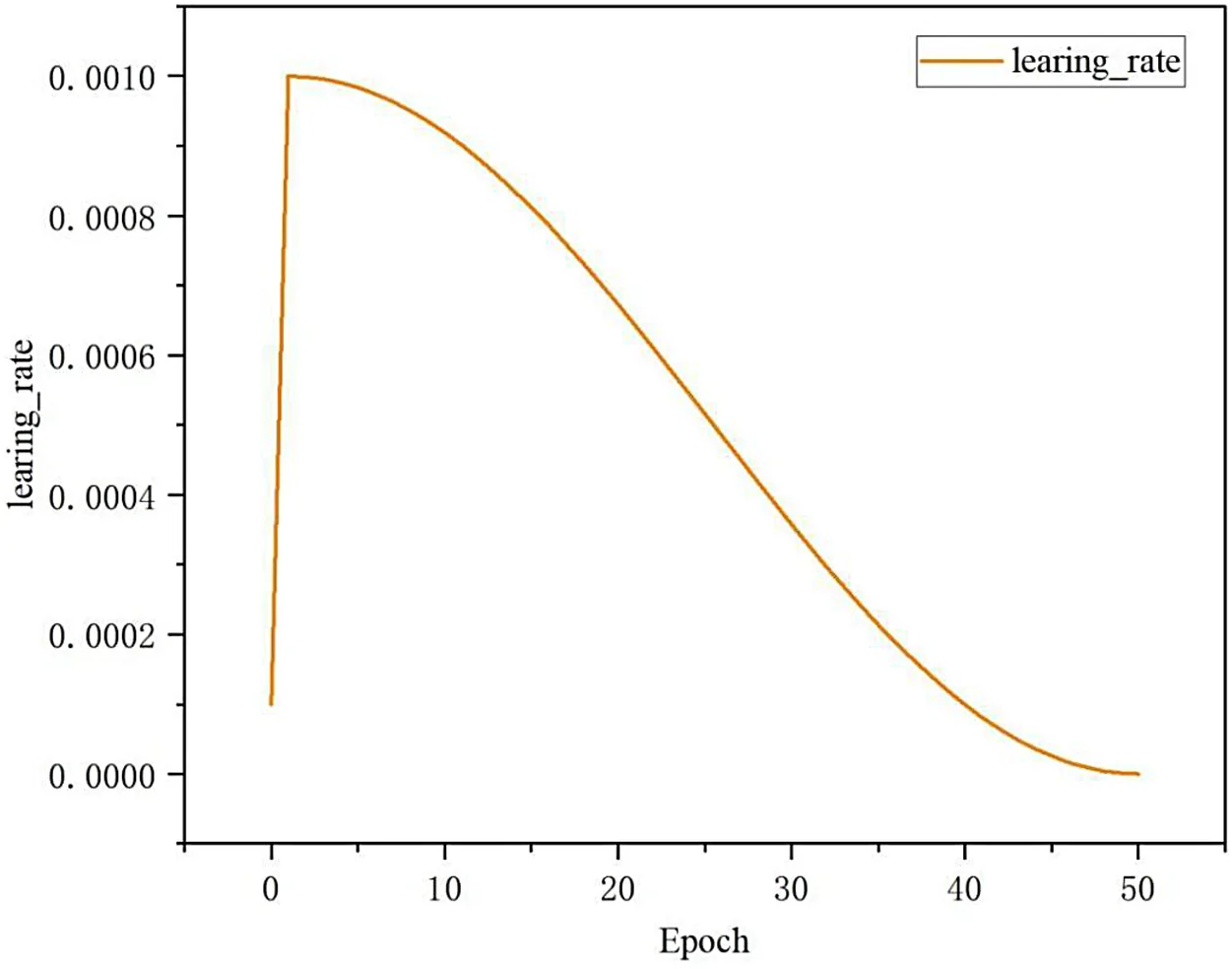
Model training learning_rate variation.

**Figure 7:**
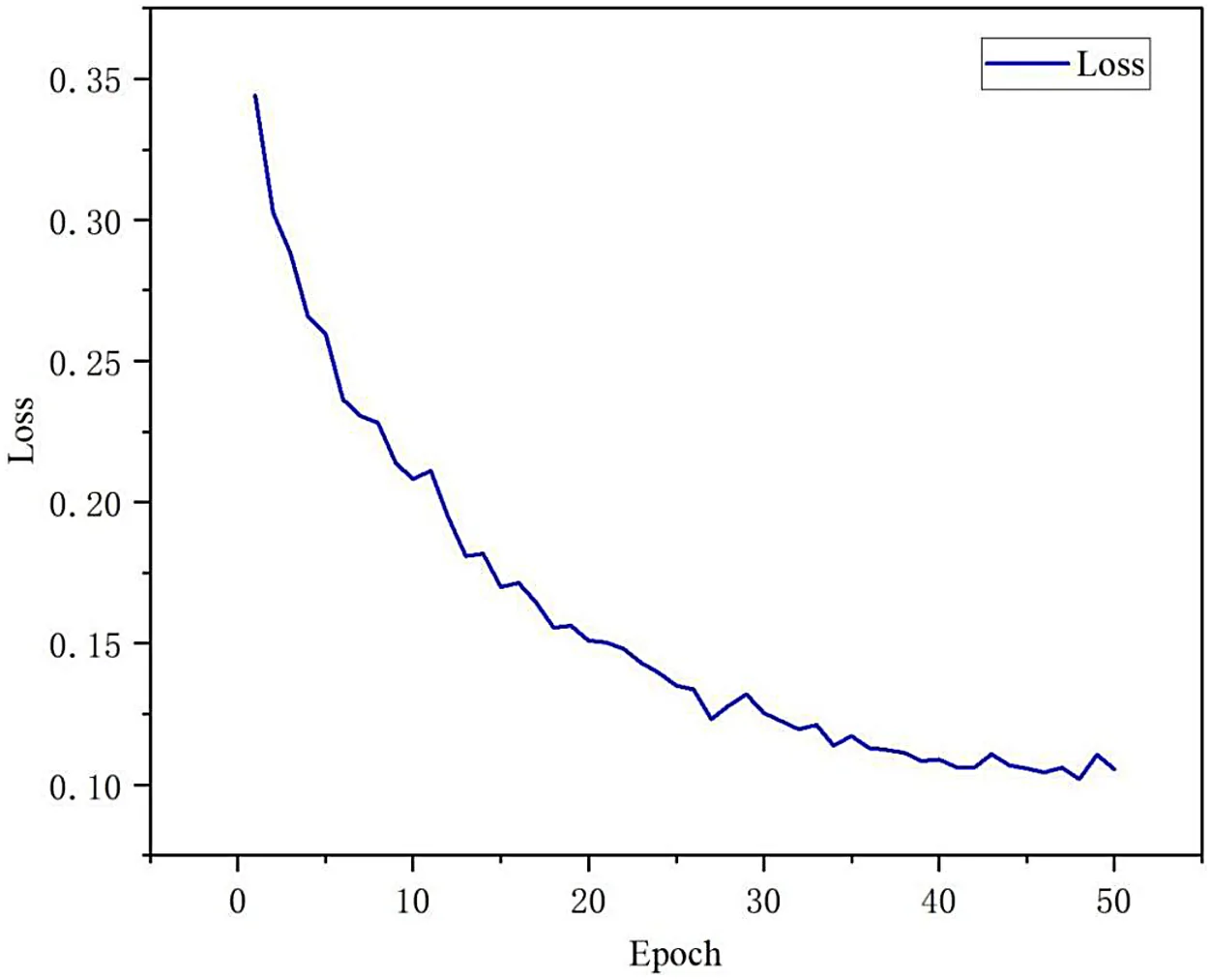
Model training loss variation.

**Table 3:** Experimental configuration.

Configurations	Values
Software version	PyTorch 1.11.0
GPU	NVIDIA RTX 3090
Optimizer	Adam
Initial learning rate	1 × 10^–3^
Learning rate decay strategy	Cosine annealing
Momentum	0.9
Batch size	16
Tranning Epoches	50
Python	3.8
CUDA	11.2
Seed	26

### 4.4 Evaluation metrics

To evaluate the segmentation performance of the proposed model, we adopts four evaluation metrics: Dice Similarity Coefficient (Dice), Sensitivity, Positive Predictive Value (PPV), and the 95th percentile of the Hausdorff Distance (HD95). These metrics are used to assess the segmentation performance of the proposed method on the whole tumor (WT), tumor core (TC), and enhancing tumor (ET) regions.

The Dice Similarity Coefficient is used to measure the similarity between two samples, with values ranging from 0 to 1, as defined in (Equation 17).

$$\mathrm{Dice}=\frac{2\mathrm{TP}}{2\mathrm{TP}+\mathrm{FP}+\mathrm{FN}}$$ (17)

A Dice coefficient closer to 1 indicates that the segmentation result is more similar to the manually annotated ground truth, and the segmentation performance is better. Conversely, a Dice coefficient closer to 0 indicates poor segmentation performance.

Sensitivity, also known as Recall, refers to the ratio of the number of samples predicted as positive by the model to the total number of actual positive samples. A higher sensitivity value indicates better accuracy in predicting positive samples, as defined in (Equation 18).

$$\mathrm{Sensitivity}=\frac{\mathrm{TP}}{\mathrm{TP}+\mathrm{FN}}$$ (18)

PPV (Precision) represents the proportion of true positive samples among all samples predicted as positive. A higher PPV value indicates greater accuracy in positive sample prediction, as defined in (Equation 19).

$$\mathrm{Precision}=\frac{\mathrm{TP}}{\mathrm{TP}+\mathrm{FP}}$$ (19)

In (Equations 17–19), TP (True Positive) denotes true positives; FN (False Negative) denotes false negatives; and FP (False Positive) denotes false positives.

To calculate the distance between the segmentation boundary generated by the model and the ground truth boundary, the Hausdorff Distance (HD) is used. The smaller the Hausdorff distance, the higher the segmentation accuracy, as defined in (Equation 20).

$$\mathrm{Hausdorff}=\max\left\{\underset{x\in X}{\max}\;\underset{y\in Y}{\min}\;d(x,y),\underset{y\in Y}{\max\;}\;\underset{x\in X}{\min}\;d(x,y)\right\}$$ (20)

Here, *y* represents the ground truth (GT), *x* denotes the predicted segmentation result, and *d*(*x*,*y*) is the Euclidean distance between *x* and *y*. In this research, HD95 is used for evaluation, which refers to the 95th percentile of the Hausdorff distance values.

### 4.5 Hybrid loss function

In brain tumor segmentation tasks, since tumor regions usually occupy only a small proportion of the image, there exists a serious class imbalance problem. Using a single conventional pixel-level loss function, such as Binary Cross-Entropy (BCE), often leads to insufficient recognition of the foreground (tumor) regions by the model. To enhance the model's sensitivity to small-volume target regions while ensuring training stability, we adopts a weighted combination of Binary Cross Entropy loss and Dice loss as the final optimization objective.

The proposed model formulates brain tumor segmentation as a three-channel multi-label segmentation task rather than a mutually exclusive multi-class segmentation task. The three output channels correspond to the whole tumor (WT), tumor core (TC), and enhancing tumor (ET), respectively. The original BraTS labels are converted into three binary masks, where WT contains labels 1, 2, and 4, TC contains labels 1 and 4, and ET corresponds to label 4. Because these regions are spatially nested and are not mutually exclusive, an independent Sigmoid activation is applied to each output channel instead of a channel-wise Softmax.

The final hybrid loss function is defined as (Equation 21).

$$L_{\mathrm{total}}=\beta L_{\mathrm{BCE}}+L_{\mathrm{Dice}}$$ (21)

Here, $L_{\mathrm{BCE}}$ denotes the Binary Cross-Entropy loss, which measures the classification error at each pixel; $L_{\mathrm{Dice}}$ represents the Dice loss, which evaluates the overlap between the predicted region and the ground truth tumor region; β represents the weight of $L_{\mathrm{BCE}}$; and $L_{\mathrm{total}}$ is the final hybrid loss function used for training.

Specifically, the Binary Cross-Entropy loss function is defined in (Equation 22).

$$L_{\mathrm{BCE}}=-\frac{1}{N}\sum_{i=1}^{N}\left[y_{i}\log\;(\;p_{i})+(1-y_{i})\log\;(1-{\hat{y}}_{i})\right]$$ (22)

where *N* denotes the total number of samples, $y_{i}$ represents the ground truth (GT), and ${\hat{y}}_{i}$ represents the predicted result.

The Dice loss function is defined in (Equation 23).

$$L_{\mathrm{Dice}}=1-\frac{2\sum_{i=1}^{N}\;p_{i}y_{i}+\varepsilon }{\sum_{i=1}^{N}\;p_{i}+\sum_{i=1}^{N}y_{i}+\varepsilon }$$ (23)

where *ε* is a small smoothing constant, set to 10^−5^, to prevent division by zero and ensure numerical stability.

The BCE loss and Dice loss are calculated independently for the WT, TC, and ET channels and then averaged across the three tumor regions. No additional region-specific or positive-class weights are used, and WT, TC, and ET contribute equally to the loss. The coefficient *β* controls the relative contribution of the BCE-loss term and is not a class-specific weight.

The weight of the Dice loss was fixed at 1, while the weight *β* of the BCE loss was selected using the validation subset. Candidate *β* values were evaluated under the same training configuration, and the corresponding validation performance is presented in Figure 8. Based on the observed validation results for the whole tumor (WT), tumor core (TC), and enhancing tumor (ET) regions, *β* was set to 1.5 for the final model. The independent testing subset was not used during this hyperparameter-selection process.

**Figure 8:**
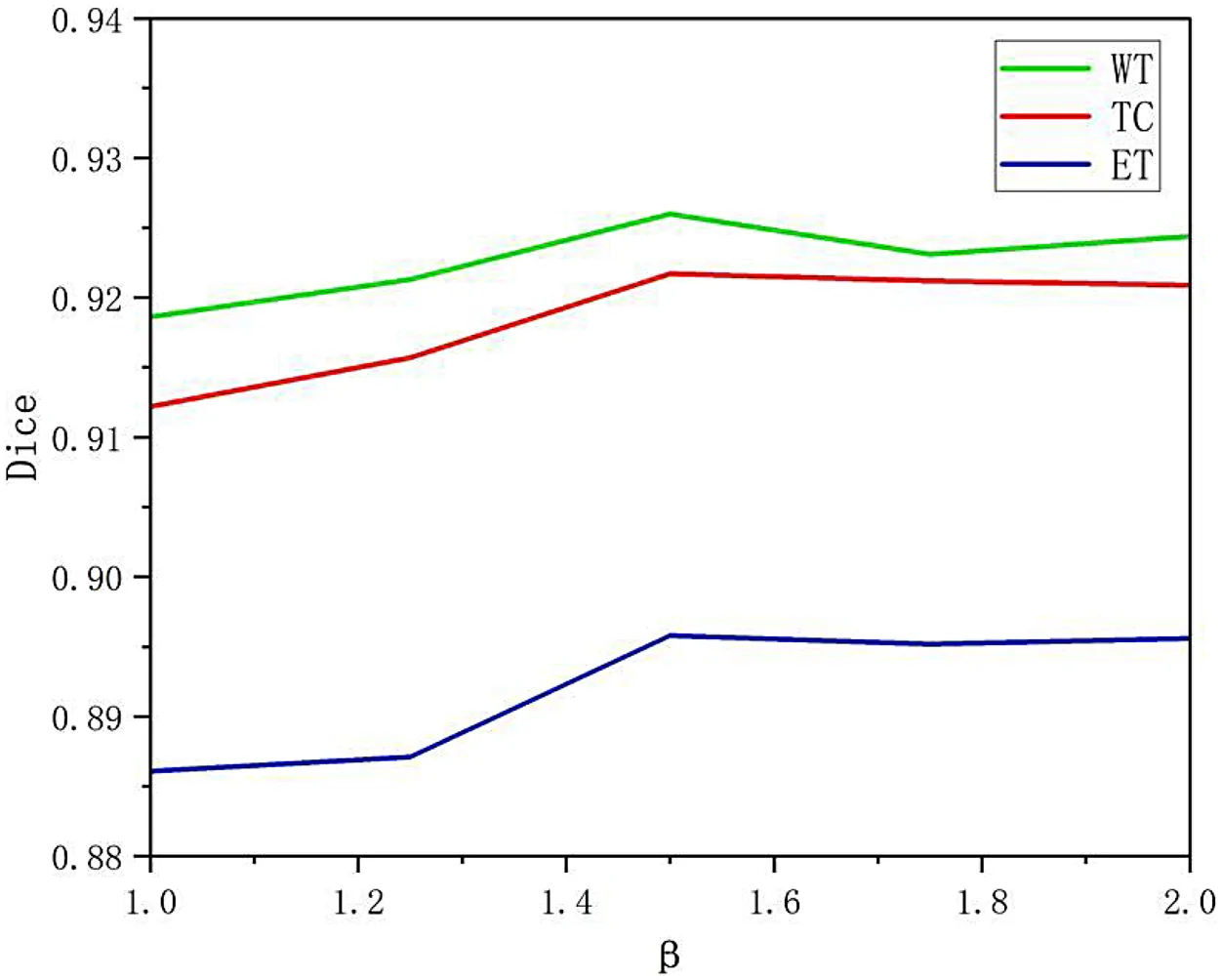
Effect of the BCE-loss weight *β* on the validation performance of MTF-Net.

### 4.6 Ablation experiment

To verify the effectiveness of the proposed components, a U-shaped network based on TransUNet was employed as the baseline structure. Specifically, MSFormer was designed to enhance multi-scale feature modeling, CEBAM was introduced to improve sparse global dependency modeling through bi-level attention, and CFIM was developed to facilitate cross-branch feature interaction between Transformer-based semantic features and convolutional structural features. All ablation experiments were conducted using the same dataset partition, random seed, optimizer, learning-rate schedule, loss function, and training settings. The individual effects of MSFormer and CEBAM were first evaluated, while the complementary contribution of CFIM was investigated within the MSFormer-enabled architecture. It should be noted that CFIM is specifically designed to fuse the features generated by the MSFormer branch and the parallel convolutional branch. Therefore, a configuration containing CFIM without MSFormer is not structurally applicable to the proposed architecture. The results are shown in Table 4.

**Table 4:** Ablation results for different module configurations on the BraTS2019 dataset.

Baseline	CEBAM	MSFormer	CFIM	Dice↑			Hausdorff95(mm)↓		
				WT	TC	ET	WT	TC	ET
√				0.852	0.834	0.810	5.570	5.545	3.490
√	√			0.858	0.850	0.826	5.534	5.517	3.450
√		√		0.876	0.865	0.840	5.481	5.481	3.409
√	√	√		0.911	0.903	0.879	5.263	5.367	3.258
√		√	√	0.905	0.896	0.870	5.307	5.395	3.298
√	√	√	√	0.926	0.921	0.896	5.123	5.310	3.203

Here, CEBAM stands for the Channel-Enhanced Bi-level Attention Module, MSFormer represents the Multi-Scale and Spatially-Aware Transformer, and CFIM denotes the Cross-Modal Feature Interaction Module.

As shown in Table 4, the baseline model achieved Dice scores of 0.852, 0.834, and 0.810 for WT, TC, and ET, respectively. Adding CEBAM alone increased the corresponding Dice scores to 0.858, 0.850, and 0.826, while adding MSFormer alone further improved them to 0.876, 0.865, and 0.840. These results indicate that both modules contribute to segmentation performance, with MSFormer providing a relatively larger improvement when evaluated individually.

When CEBAM and MSFormer were jointly incorporated without CFIM, the Dice scores increased to 0.911, 0.903, and 0.879 for WT, TC, and ET, respectively. Compared with this configuration, the complete MTF-Net further improved the Dice scores by 0.015, 0.018, and 0.017, while reducing the corresponding HD95 values by 0.140, 0.057, and 0.055 mm. This comparison demonstrates the additional contribution of CFIM to feature fusion and boundary delineation.

Furthermore, compared with the MSFormer + CFIM configuration, introducing CEBAM increased the Dice scores by 0.021, 0.025, and 0.026 for WT, TC, and ET, respectively, and reduced the corresponding HD95 values by 0.184, 0.085, and 0.095 mm. The complete model achieved the best overall results among all evaluated configurations, suggesting that CEBAM, MSFormer, and CFIM provide complementary contributions to tumor-region segmentation.

It should be noted that the ablation results in Table 4 were obtained from a single run using a fixed random seed of 26. No repeated-seed experiments, confidence intervals, or paired statistical tests were performed. Therefore, the reported differences represent descriptive numerical changes under the specified experimental setting and should not be interpreted as statistically significant improvements.

### 4.7 Experimental results

To further verify the effectiveness of the proposed method in multi-modal brain tumor segmentation, we compare MTF-Net with several representative segmentation models on the BraTS2019 and BraTS2021 datasets. The experimental results are shown in Tables 1, 2, with corresponding visual segmentation results displayed in Figures 9, 10. The comparison includes both 2D and 3D segmentation methods to provide a broader reference for evaluating MTF-Net. However, 2D and 3D methods differ in input dimensionality and spatial-context modeling. Therefore, the comparison with 3D methods should be regarded as a benchmark-level reference based on common tumor regions and evaluation metrics, rather than as a strictly controlled demonstration that the proposed 2D architecture is superior to volumetric approaches.

**Figure 9:**
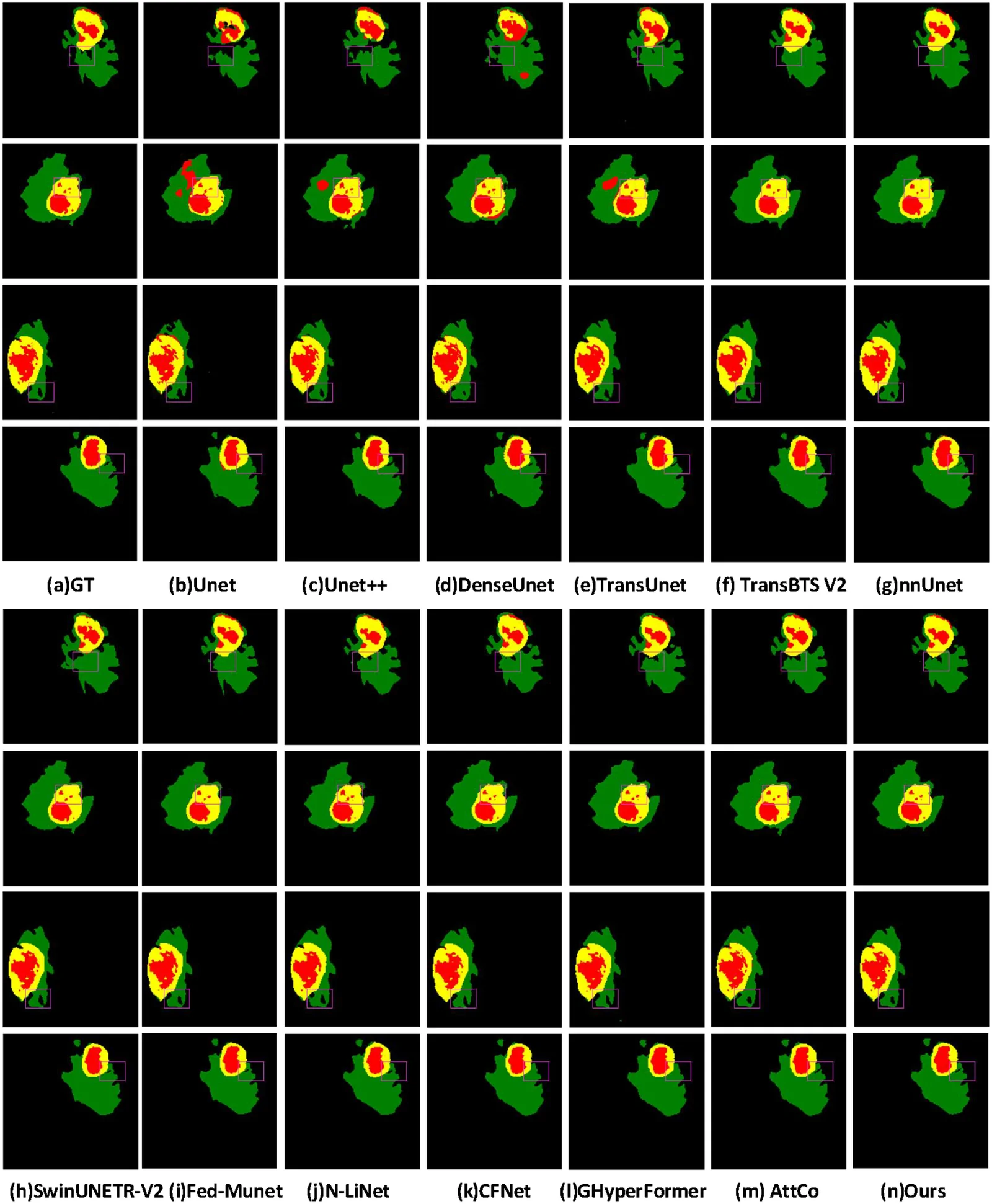
Visual comparison of segmentation results on BraTS2019 dataset: **(a)** GT; **(b)** Unet; **(c)** Unet++; **(d)** DenseUnet; **(e)** TransUnet; **(f)** TransBTSV2; **(g)** nnUnet; **(h)** SwinUNETR-V2; **(i)** Fed-MUnet; **(j)** N-LiNet; **(k)** CFNet; **(l)**GHyperFormer; **(m)** AttCo; **(n)** ours.

**Figure 10:**
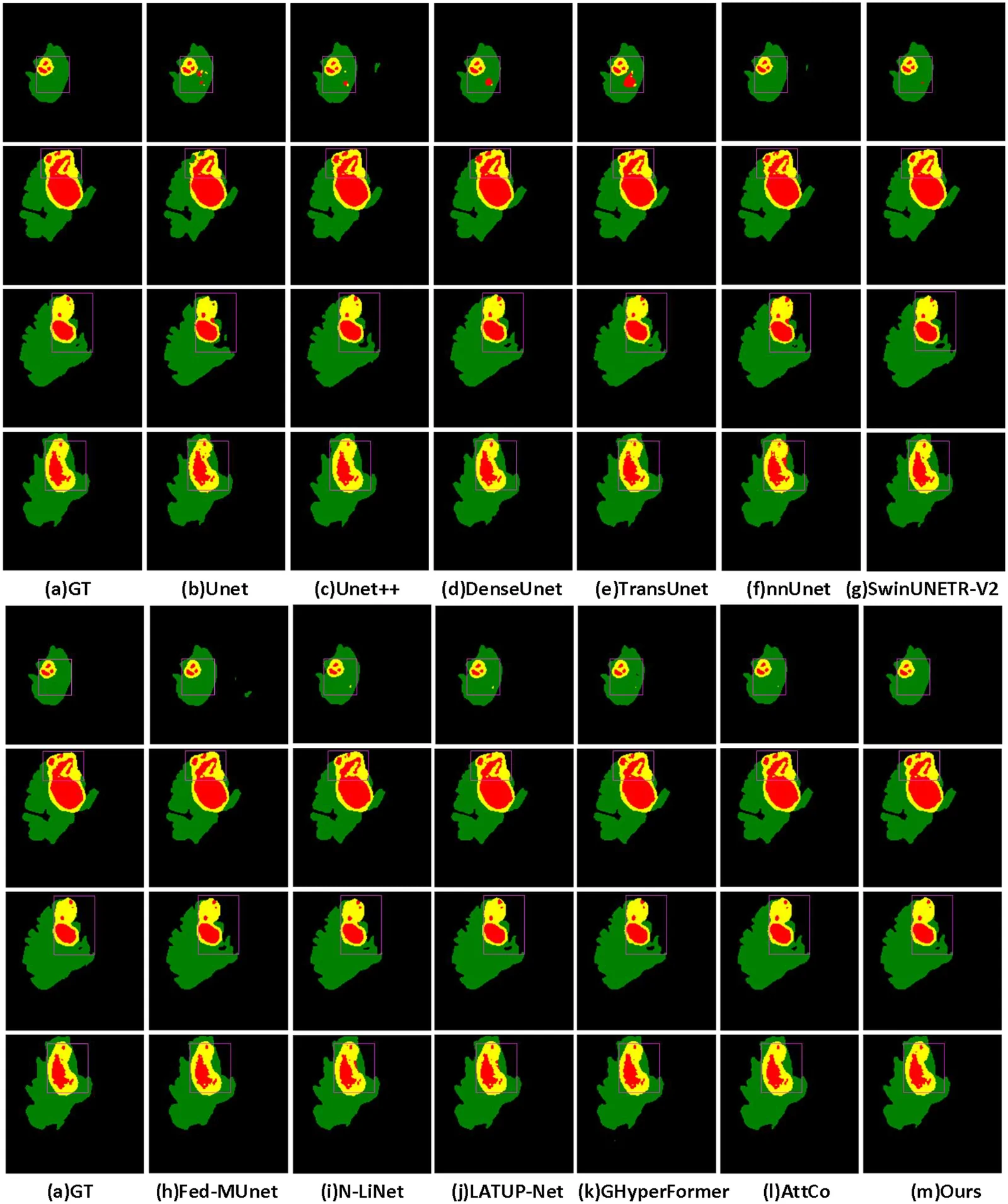
Visual comparison of segmentation results on BraTS2021 dataset: **(a)** GT; **(b)** Unet; **(c)** Unet++; **(d)** DenseUnet; **(e)** TransUnet; **(f)** nnUnet; **(g)** SwinUNETR-V2; **(h)** Fed-MUnet; **(i)** N-LiNet; **(j)** LATUP-Net; **(k)** GHyperFormer; **(l)** AttCo; **(m)**Ours.

#### 4.7.1 Comparison with BraTS2019 results

On the BraTS2019 dataset, MTF-Net achieved Dice scores of 0.926, 0.921, and 0.896 for the WT, TC, and ET regions, respectively, which were higher than those of the other evaluated methods in Table 1. MTF-Net also obtained the highest Precision and Sensitivity values for all three tumor regions among the evaluated methods. In terms of HD95, MTF-Net achieved the lowest values for WT and TC. However, Fed-MUNet obtained a slightly lower ET HD95 value than MTF-Net. These results indicate that MTF-Net provides competitive and relatively balanced segmentation performance across the evaluated metrics, although it does not achieve the best result for every individual metric.

Several representative samples were selected from the BraTS2019 testing subset for qualitative comparison, as shown in Figure 9, where each row represents one patient case. U-Net exhibits obvious over-segmentation problems in multiple cases, especially in the first and third rows, where the whole-tumor region shows excessively expanded boundaries and blurred contours. In addition, confusion can be observed between the enhancing-tumor and necrotic/non-enhancing tumor regions. U-Net++ shows improvements over U-Net in boundary constraint, but over-segmentation of the enhancing tumor region can still be observed in the second- and fourth-row samples. DenseUNet demonstrates certain advantages in maintaining structural continuity, and its prediction of the tumor core region is relatively compact. However, in some cases, such as the fourth row, it still fails to accurately distinguish between the necrotic and enhancing regions. TransUNet shows clearer boundary delineation in the TC and ET regions in several cases, but missegmentation of the ET region occurs in the second row. TransBTSv2 performs stably overall, with relatively complete structural modeling of the edema and core regions. Some tumor-subregion boundaries are close to the ground truth, although boundary deviation can still be observed in the ET region in the third row. nnU-Net generally preserves the overall shape of the tumor region, although local deviations can still be observed around the TC and ET boundaries in some cases. SwinUNETR-V2 produces relatively smooth and coherent tumor contours, but minor boundary deviations remain in several ET predictions. Fed-MUNet produces predictions that are relatively close to the ground-truth annotations for the three subregions in several displayed cases, but shows over-segmentation in the whole-tumor region in the first row. N-LiNet produces relatively compact WT and TC predictions, although some fine ET details are not fully recovered in the displayed cases. CFNet and GHyperFormer can effectively segment the WT and TC regions, although some ET details in the displayed examples appear less closely aligned with the ground-truth annotations than those produced by MTF-Net. AttCo also provides relatively stable segmentation of the WT and TC regions, while minor discrepancies can still be observed around some ET boundaries.

In the displayed examples, MTF-Net generally produces relatively compact tumor contours and predictions that are visually close to the ground-truth annotations. In particular, the enhancing-tumor boundaries in several displayed cases appear relatively coherent compared with those produced by several other evaluated methods. Nevertheless, these qualitative observations are limited to the selected examples and should not be interpreted as evidence that MTF-Net is superior in every patient case. Together with the quantitative results in Table 1, the visual comparisons suggest that MTF-Net has competitive boundary delineation and tumor-subregion segmentation capabilities.

#### 4.7.2 Comparison with BraTS2021 results

On the BraTS2021 dataset, MTF-Net achieved Dice scores of 0.918, 0.923, and 0.901 for the WT, TC, and ET regions, respectively. These values were higher than those of the evaluated classical 2D models, including U-Net, U-Net++, DenseUNet, and TransUNet. Among the evaluated 3D and hybrid methods, MTF-Net achieved the highest Dice scores for TC and ET, whereas GHyperFormer obtained a slightly higher WT Dice score of 0.919. Therefore, MTF-Net demonstrated competitive and relatively balanced performance across the three tumor regions.

Among the other evaluated methods, nnU-Net achieved WT, TC, and ET Dice scores of 0.888, 0.851, and 0.796, respectively. SwinUNETR-V2 and N-LiNet achieved corresponding Dice scores of 0.910, 0.900, and 0.878, and 0.910, 0.897, and 0.875, respectively. AttCo achieved Dice scores of 0.916, 0.908, and 0.872, including the second-highest TC Dice score among all evaluated methods.

For Precision, MTF-Net achieved the highest TC value of 0.953. Its WT Precision of 0.924 was lower than those of GHyperFormer, Fed-MUNet, and AttCo, while its ET Precision of 0.921 was lower than that of Fed-MUNet. For Sensitivity, MTF-Net achieved the highest value for TC, whereas GHyperFormer and AttCo obtained higher values for WT. AttCo achieved the highest ET Sensitivity of 0.892, followed by MTF-Net with 0.884. In terms of HD95, MTF-Net achieved the lowest values for WT, TC, and ET among the evaluated methods. These results indicate that MTF-Net provides competitive and relatively balanced segmentation performance across the three tumor regions, although it does not achieve the best result for every individual metric.

Figure 10 presents the visual segmentation results of different models on the BraTS2021 dataset. The evaluated methods generally captured the principal tumor regions, although differences in local boundary delineation and small enhancing-tumor structures can be observed in the displayed cases. In several displayed cases, MTF-Net produces relatively clear enhancing-tumor contours and predictions that are visually close to the ground-truth annotations. However, these qualitative observations are limited to the selected examples and should not be interpreted as evidence that MTF-Net is superior in every patient case. Together with the quantitative results in Table 2, the visual comparisons suggest that MTF-Net achieves competitive segmentation performance among the evaluated 2D, 3D, and hybrid brain tumor segmentation methods.

Overall, MTF-Net achieves competitive segmentation performance compared with the evaluated 2D and 3D brain tumor segmentation methods.

### 4.8 Statistical analysis

To further evaluate the robustness of MTF-Net, patient-level statistical analysis was performed on the BraTS2019 and BraTS2021 testing cohorts, as shown in Table 5. Unlike the overall Dice scores reported in Tables 1, 2, the following analysis calculates Dice values for each individual patient and reports the results as mean ± standard deviation (SD). In addition, the Wilcoxon signed-rank test was conducted to evaluate whether the performance improvements over the baseline methods were statistically significant.

**Table 5:** Patient-level statistical analysis of MTF-Net on BraTS2019 and BraTS2021 datasets.

Dataset	Region	Dice (mean ± SD)	*p*-value
BraTS2019	WT	0.926 ± 0.0170	<0.05
	TC	0.921 ± 0.0247	<0.05
	ET	0.896 ± 0.0261	<0.05
BraTS2021	WT	0.918 ± 0.0428	<0.05
	TC	0.923 ± 0.0314	<0.05
	ET	0.901 ± 0.0637	<0.05

### 4.9 Complexity comparative analysis

To evaluate the model size and theoretical computational complexity of the proposed method, we compared MTF-Net with representative 2D and 3D segmentation models in terms of the number of trainable parameters and floating-point operations, as shown in Table 6.

**Table 6:** Comparison of models with params and FLOPs.

Models	Params(M)	FLOPs(G)
U-net (5)	69.71	28.46
TransUnet (6)	109.07	43.51
TransBTSV2 (24)	32.99	333.09
Mmformer (25)	106.00	748.00
CFNet (28)	53.50	165.47
GHyperFormer (29)	13.18	320.17
Ours(MTF-Net)	100.28	79.30

MTF-Net contains 100.28 M trainable parameters and requires 79.30 GFLOPs for an input consisting of four MRI modalities with a spatial resolution of 160 × 160. Compared with representative 2D segmentation models, MTF-Net has fewer parameters than TransUNet, which contains 109.07 M parameters. However, its computational cost is higher than those of U-Net and TransUNet. This increase is mainly associated with the parallel multi-scale feature extraction, cross-branch feature interaction, and global context modeling incorporated into the proposed architecture. Compared with representative 3D and hybrid segmentation methods, MTF-Net exhibits substantially lower computational complexity. Specifically, its FLOPs are approximately 76.19%, 89.40%, 52.08%, and 75.23% lower than those of TransBTSV2, mmFormer, CFNet, and GHyperFormer, respectively. Although GHyperFormer has a smaller parameter count, its FLOPs are considerably higher than those of MTF-Net.

As illustrated in Figure 11, MTF-Net achieves the highest average Dice score among the compared methods on the BraTS2019 dataset while maintaining moderate theoretical computational complexity. These results suggest that MTF-Net achieves a favorable balance between segmentation accuracy, model capacity, and computational cost, particularly in comparison with computationally intensive 3D and hybrid segmentation models. Table 6 Comparison of models with Params and FLOPs. Furthermore, we evaluated the practical resource requirements of MTF-Net during training. Under the experimental environment equipped with an NVIDIA RTX 3,090 GPU (24 GB memory), the complete training process required approximately 10 h, and the maximum GPU memory consumption was approximately 20 GB. These results demonstrate that although MTF-Net incorporates multiple feature interaction mechanisms, its computational time and memory requirements remain acceptable for practical medical image segmentation applications.

**Figure 11:**
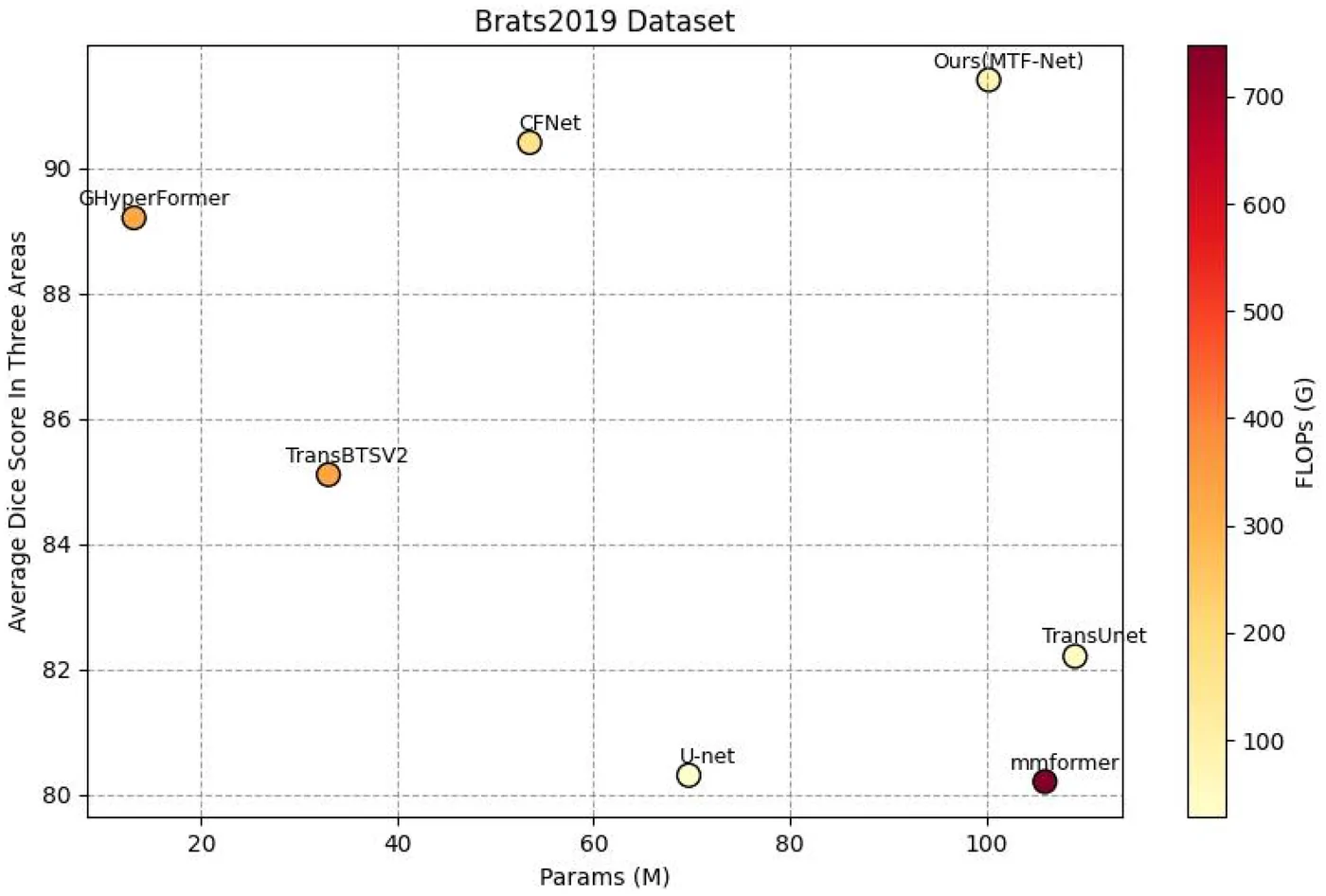
Model performance comparison chart.

## 5 Conclusion

To address the issues of strong tumor region heterogeneity, blurry boundaries, and variable morphology in multimodal MRI brain tumor segmentation, we propose a brain tumor segmentation network based on multi-scale Transformer and feature fusion enhancement (MTF-Net). MTF-Net constructs an efficient encoder-decoder structure through the MSFormer, CEBAM, and CFIM modules to enhance multi-scale feature expression and the ability to capture long-range dependency features. The MSFormer module combines multi-scale convolutional receptive fields with spatially-aware MLP, effectively improving local context and long-range dependency modeling. The CEBAM module uses a dual-layer sparse attention mechanism and channel enhancement strategy to achieve structure-sensitive modeling. The CFIM module fuses features from different modalities and layers through multi-branch convolution and coordinate attention mechanisms, thus narrowing the semantic gap between the encoder and decoder. During network training, a redesigned hybrid loss function is introduced to handle class imbalance issues. Experimental results demonstrate that MTF-Net achieves competitive performance compared with existing methods on both the BraTS2019 and BraTS2021 datasets. Moreover, the ablation results support the contributions of the designed modules to the overall segmentation performance of the network under the reported experimental setting.

Although MTF-Net demonstrates competitive segmentation performance on the BraTS2019 and BraTS2021 datasets, several limitations remain. First, both datasets are derived from the same BraTS benchmark family. Although experiments on these two independent cohorts provide preliminary evidence of model robustness, further validation on external clinical datasets collected from different institutions is required to comprehensively evaluate the generalization ability of the proposed method. Moreover, clinical factors such as expert reader evaluation, uncertainty estimation, robustness assessment, and prospective clinical validation were not investigated. Therefore, further studies involving multi-center validation and comprehensive clinical evaluation are required before potential clinical translation.

In addition, the quantitative results were obtained from a single run using a fixed random seed of 26. Case-level metric distributions, confidence intervals, repeated-seed variability analyses, and paired statistical tests were not performed. Therefore, the observed numerical differences should not be interpreted as statistically significant, and their robustness across different random seeds and data partitions remains to be further evaluated.

The published comparison results may also have been obtained using different data partitions, preprocessing procedures, training configurations, and evaluation protocols. Therefore, the numerical comparisons should be interpreted as benchmark-level references rather than as strictly controlled evidence of state-of-the-art superiority.

Furthermore, the current model processes axial slices independently and does not explicitly use information from adjacent slices. Therefore, some three-dimensional contextual information may be lost, and spatial inconsistency may occur between neighboring slice predictions. The proposed method should thus be regarded as a 2D slice-based segmentation framework rather than a complete volumetric segmentation solution. Future work will investigate repeated-seed and case-level statistical analyses, as well as 2.5D or 3D extensions that incorporate adjacent-slice information while maintaining segmentation accuracy.

## Data Availability

The original contributions presented in the study are included in the article/Supplementary Material, further inquiries can be directed to the corresponding author.
